# Financing Long-term Care: Some Ideas From Switzerland: Comment on "Financing Long-term Care: Lessons From Japan"

**DOI:** 10.15171/ijhpm.2019.83

**Published:** 2019-10-04

**Authors:** Martin Eling

**Affiliations:** Institute of Insurance Economics, School of Finance, University of St. Gallen, St. Gallen, Switzerland.

**Keywords:** Long-term Care, Social Security, Insurance, Switzerland

## Abstract

Ikegami reviews the implementation of mandatory long-term care insurance systems in Germany and Japan, which are organized as pay-as-you-go systems. I propose to go one step further and implement a multi-pillar, mandatory and voluntary long-term care financing system, which combines pay-as-you-go with capital-funded elements. The proposal is based on the observation that Switzerland has implemented a three-pillar system for financing retirement provisions that can be adapted to finance long-term care in a fair and sustainable way.

## Motivation

Expenditure on long-term care will rise significantly in relation to the gross domestic product in many industrialized countries in the next decades, making its financing one of the most pressing societal challenges. These costs must be financed, whether through dedicated savings or capital-funded insurance schemes or through taxes, pay-as-you-go (a separate system where beneficiaries are directly financed by the working population^1^) or out-of-pocket payments.

Substantial uncertainties regarding the potential need, intensity and duration of long-term care provide compelling arguments for a risk transfer (ie, a risk financing via an insurance scheme) both at the individual and societal levels. Nevertheless, corresponding insurance solutions have rarely been used. Many countries rely on a mix of tax-financed means-tested schemes and out-of-pocket expenses, while only a few countries like Japan and Germany have established universal social insurance schemes, typically organized as pay-as-you-go systems. In this context, Ikegami^2^ reviews the German and Japanese systems and elaborates upon some of their intended and unintended consequences of the system introduction. Although the article discusses some of the critical aspects, Ikegami concludes that implementing a public long-term care insurance system is beneficial; resources are more equitably allocated based on objective eligibility criteria and the insurance contributions are more accepted by the population than higher taxes.

I propose to go even one step further and implement a multi-pillar system for financing long-term care. My proposal is based on the observation that Switzerland has implemented a three-pillar system for retirement provisions, which can also be used to finance long-term care in a sustainable and equitable manner. In light of the imminent demographic change, some elements of the expected costs should in my view be put aside as savings or capital-funded insurance schemes, if large intergenerational transfers are to be avoided. Given the foreseeable cost pressure in the coming decades, both pay-as-you-go and capital-funded systems might be necessary to finance long-term care.

## A Global Review

Reviewing the financing of long-term care globally, two third of the financing is done through taxes and out-of-pocket payments; the remaining third of the financing is organized in social security systems such as those implemented in Japan or Germany.^3^ These tend to be pay-as-you-go systems, meaning that today’s working generation finances the costs of today’s beneficiaries. An alternative to this redistribution from current workers to beneficiaries might be a capital-funded scheme where the population accumulates assets for their own future risk.

Traditionally, pay-as-you-go systems worked well, because the working population was capable of carrying the necessary financing. However, with the demographic change and the increasing old age quotient, this system is approaching its limit; the working population decreases while the number of beneficiaries increases. This is especially true for long-term care, given that the old age dependency ratio (number of people above 80 years old per 100 population) is projected to triple from 4% in 2010 to 12% in 2050 in the Organisation for Economic Co-operation and Development (OECD) countries.^4^ Most of the people who need long-term care are older than 80 years of age, making the development of the old age dependency ratio an important predictor for future long-term care financing needs.

There have been diverging trends such as an increase of public coverage of long-term care expenditures in France, Japan, Spain, and Korea, but a decrease in Germany, Sweden, and the Netherlands. Some countries such as Germany have introduced capital-funded elements, while other countries purely rely on tax-based means-tested financing of long-term care costs. Overall, it is unclear how the optimal financing for long-term care should look. It is also likely that there is not one optimal model; the best model depends on economic, social, cultural and demographic factors of the respective country. One simple example for this is that a pay-as-you-go financing scheme will work well in countries with growing populations and economies, but not so well in countries that are stagnating. Many industrialized countries are, however, facing comparable demographic, socio-economic and political challenges, with the baby-boomer generation retiring now, resulting in greater pressure on social security in the coming decades.

There are manifold reasons why voluntary insurance and savings solutions are hardly used. Limited knowledge, a low value of consumption while in care as well as the availability of public and private substitutes were cited as reasons for the non-existence of private markets for long-term care financing. A voluntary insurance system organized in a free market is thus very likely not going to work on a broader scale. A mandatory system is thus needed for financing long-term care.

## The Situation in Switzerland

In the 1980s, Switzerland complemented its social security system with capital-funded elements, resulting in a three-pillar system for retirement provisions. The first pillar is a tax financed pay-as-you-go insurance regime that secures substantial needs. The second pillar comprises individual capital-funded occupational retirement plans designed to secure an appropriate living standard. The third pillar is tax subsidized voluntary private retirement savings that can finance additional needs beyond what the legislator considers an appropriate living standard. Many international studies, for example by the OECD,^5^ the World Bank,^6^ or other international organisations,^7^ have concluded that Switzerland has a very good retirement system; combining pay-as-you-go and capital-funded elements makes the system more diversified than other retirement systems that rely on only one of these two elements. More than 50% of the retirement provisions now comes out of a capital-funded pension sector that has accumulated more than $US900 billion, a stock that is bigger than the gross domestic product of Switzerland (approximately $US700 billion in 2018).^8^ Also the third pillar has accumulated more than $US100 billion.^9^

While the Swiss financing of the retirement provisions is often considered a role model for other countries, the financing of long-term care has not been subject of a reform and is financed via the mandatory health insurance scheme, taxes at the municipal level and out-of-pocket expenses. Switzerland’s long-term care costs are projected to double from $US16 billion today to $US32 billion per year in 2050, an increase from 2.4% of the gross domestic product to around 4.8%.^10^ The out-of-pocket payments personal contributions of those in need of care already account for a high proportion of total financing in Switzerland compared with other countries (30%, while the average internationally is only 13.5%).^3^ If this status quo is maintained, the financial burden on those in need of care and on the municipals will reach the limits of feasibility. Accordingly, the sustainability of long-term care financing is not given, which has resulted in several recent political discussions.

My proposal is to use the experiences from the three-pillar system for the retirement provisions and implement a pay-as-you-go system to cover a minimum standard for long-term care, a capital accumulation to cover a decent standard for long-term care protection and voluntary private insurance solutions for those who want more coverage. The first and the second pillars need to be mandatory for the above discussed reasons; the third pillar should be voluntary, but can be tax-subsidized if the government wants to encourage or reward additional savings.

## Conclusion and Recommendations

A discussion of alternative financing models for long-term care costs must be done based on the criteria of equity and efficiency. For equity especially the social justice between rich and poor and the intergenerational fairness between young and old parts of the population are in focus. For efficiency the effect of a reform on the current economy, the potential incentive effects, and the sustainability of a reform need to be considered. Given these criteria, it is surprising that the use of capital-funded insurance systems was never seriously considered for long-term care financing. Very likely, this is because the implementation creates a double burden for the now-working generation that not only has to pay the beneficiaries, but also has to accumulate capital for their own future needs. Complementing the existing system thus needs to be done in a prudent way that does not overload the capacity of the working population. Low income people that cannot afford additional savings need to be supported by social politics (eg, via subsidies), leading to some redistribution between rich and poor parts of the population. The alternative to add nothing to the current system will, however, result in excessive burdens for the future generation, which seems problematic from an intergenerational point of view. We thus recommend supplementing the current financing system with some capital-funded insurance or savings solution.

The discussion clearly indicates the enormous complexity of social policy. Changing the system moves the entire setup of social policy, especially redistribution between poor and rich and between the generations. A discussion of possible additions to the current system for ensuring sustainable financing seems necessary in many industrialized countries with aging populations.

## Ethical issues

Not applicable.

## Competing interests

Author declares that he has no competing interests.

## Author’s contribution

ME is the single author of the paper.
